# Transkranielle Gleichstromstimulation zur Verbesserung der Trainingseffektivität bei chronischer Aphasie nach Schlaganfall – wie gelingt die Studienrekrutierung Betroffener?

**DOI:** 10.1007/s00115-023-01572-7

**Published:** 2024-01-04

**Authors:** Nina Unger, Benjamin Stahl, Robert Darkow, Veronika Scholz, Isabel Weinmar, Johanna Schmidt, Caterina Breitenstein, Marcus Meinzer, Tanja Grewe, Agnes Flöel

**Affiliations:** 1 Klinik und Poliklinik für Neurologie, Universitätsmedizin Greifswald, Ferdinand-Sauerbruch-Straße, 17475 Greifswald, Deutschland; 2 Fakultät Naturwissenschaften, Medical School Berlin, Berlin, Deutschland; 3 Max-Planck-Institut für Kognitions- und Neurowissenschaften, Leipzig, Deutschland; 4 Institut Logopädie, FH Joanneum, Graz, Österreich; 5 Klinik für Neurologie mit Institut für Translationale Neurologie, Universität Münster, Münster, Deutschland; 6 Abt. Technik & Gesundheit für Menschen, Studiengang Logopädie, Jade Hochschule, Oldenburg, Deutschland

**Keywords:** Aphasie, Klinische Studie, Logopädie, Rekrutierung, Transkranielle Hirnstimulation, Aphasia, Clinical trial, Speech therapy, Patient recruitment, Transcranial direct current stimulation

## Abstract

**Einleitung/Hintergrund:**

DC_TRAIN_APHASIA ist eine multizentrische, randomisiert-kontrollierte Studie, die seit November 2019 unter Federführung der Universitätsmedizin Greifswald durchgeführt wird (ClinicalTrials.gov Identifier: NCT03930121). Die Studie untersucht, ob adjuvante transkranielle Gleichstromstimulation („transcranial direct current stimulation“, tDCS) den Erfolg einer 3‑wöchigen intensiven Sprachtherapie bei chronischer Aphasie steigern kann.

**Material und Methode:**

Bis Ende 2024 sollen bundesweit 130 Patient:innen eingeschlossen werden. Die Entwicklung innovativer Rekrutierungsstrategien stellt seit Beginn der Studie eine Herausforderung dar. Neben gängigen Rekrutierungsmethoden wie der direkten Ansprache von Menschen mit Aphasie in Kliniken, Logopädiepraxen, Rehabilitationseinrichtungen und Selbsthilfegruppen wurden Radiowerbespots, Fernsehbeiträge und Auftritte in sozialen Medien erprobt.

**Zwischenergebnisse:**

Bis zum aktuellen Zeitpunkt konnten 110 Patient:innen in die Studie eingeschlossen werden. Zum größten kurzzeitigen Rücklauf führte die Rekrutierung über einen Fernseh- bzw. Radiobeitrag. Den größten langfristigen Rücklauf ergab die Rekrutierung über Logopädie- und Neurologiepraxen, Selbsthilfegruppen und soziale Medien. Teilnehmer:innen berichteten als „Testimonials“ positiv von der Sprachtherapie und der Anwendung von tDCS, die sich als gut verträglich erwies.

**Diskussion:**

Die multizentrische Studie DC_TRAIN_APHASIA prüft die Wirksamkeit von tDCS als adjuvante Applikation für intensive Sprachtherapie bei chronischer Aphasie. Die vorliegende Übersicht soll künftigen Studien als Leitfaden zur Rekrutierung von Stichproben dienen, die Menschen mit eingeschränkten kommunikativen Fähigkeiten umfassen.

## Abstract (en)

**Introduction/background:**

DC_TRAIN_APHASIA is an ongoing multicenter, randomized controlled trial, conducted since November 2019 under the lead of the University Medicine Greifswald (ClinicalTrials.gov Identifier: NCT03930121). The study seeks to determine whether adjuvant transcranial direct current stimulation (tDCS) can increase the effectiveness of a 3‑week treatment with intensive speech-language therapy in chronic post-stroke aphasia.

**Material and method:**

Until the end of 2024, a total of 130 patients are to be included in Germany. Recruitment has been a challenge throughout the study and substantial efforts went into devising innovative recruiting approaches. Standard recruitment strategies were used, such as directly approaching people with aphasia in clinical settings, inpatient and outpatient language rehabilitation facilities, and patient support and advocacy groups, alongside more innovative techniques including radio commercials, dissemination of study information via national television and social media platforms.

**Provisional results:**

Up until now, 110 patients have been included into the study. The largest short-term response was achieved via television and radio. The largest long-term response was obtained through recruitment via logopaedic and neurological facilities, patient support groups, and social media. Participants served as “testimonials”, expressing that they were satisfied with the therapy and the tDCS application.

**Discussion:**

The multicenter study DC_TRAIN_APHASIA aims to provide evidence on tDCS as an adjuvant application to increase the effect size of intensive speech-language therapy in chronic post-stroke aphasia. The present review may guide future studies in recruiting samples that involve people with impaired communicative abilities.

## Einleitung/Hintergrund

Intensive Sprachtherapie kann im chronischen Stadium einer Aphasie zu Verbesserungen der Kommunikation in alltagsnahen Situationen führen. Dies konnte in einer multizentrischen randomisiert-kontrollierten Studie von Breitenstein et al. [3] anhand einer Stichprobe von 156 Personen gezeigt werden. Die 3‑wöchige Intensivtherapie bestand aus mind. 15 Übungsstunden pro Woche in der Interventionsgruppe und umfasste sprachsystematische und kommunikativ-pragmatische Methoden, ergänzt durch computergestützte Verfahren zum Eigentraining. Die erzielten Fortschritte blieben auch 6 Monate nach Behandlungsende bestehen [3]. Verglichen wurden die Ergebnisse nach der Intensivtherapie (Interventionsgruppe) mit einer entsprechend langen Wartezeit ohne intensive Sprachtherapie (Kontrollgruppe). Die Effektstärke des Gruppenvergleichs lag im mittleren Bereich (Cohen’s d = 0,58); die numerische Punktwertänderung von vor zu nach der Therapie fiel bei den meisten Teilnehmenden moderat aus [3].

Parallel dazu legten mehrere Proof-of-concept-Studien nahe, dass transkranielle Gleichstromstimulation („transcranial direct current stimulation“, tDCS) ein einfach anzuwendender, gut verträglicher und kostengünstiger Ansatz zur Wirksamkeitssteigerung von Sprachtherapie bei chronischer Aphasie nach Schlaganfall sein könnte [2, 4–7]. Nach mehreren Arbeiten an kleinen Kollektiven von 10 (Baker et al. [2]) bzw. 8 (Fridriksson et al. [6]) Teilnehmenden mit chronischer Aphasie, wurde in einer größeren randomisiert-kontrollierten Studie mit 74 Patient:innen mit chronischer Aphasie aus derselben Arbeitsgruppe [7] berichtet, dass ein Benenntraining mit a‑tDCS über temporalen Bereichen der linken Hemisphäre zu einer 70 % höheren Benennleistung im Vergleich zu sham-tDCS führte.

Eine randomisiert-kontrollierte Phase-II-Studie von Meinzer et al. [13] mit 26 Patient:innen mit chronischer Aphasie lieferte Hinweise auf eine erhöhte Wirksamkeit eines intensiven computergestützten Sprachtrainings durch a‑tDCS über dem linken primärmotorischen Kortex (M1) gegenüber sham-tDCS. Es zeigten sich für den Gruppenvergleich (a-tDCS vs. sham-tDCS) nach Abschluss des Trainings sowie im Follow-up nach 6 Monaten hohe Effektstärken, allerdings vorwiegend für das Benennen von Objekten. Alltagsnahe Kommunikation wurde ausschließlich durch Fragebögen erfasst.

Zusammengefasst deuten die vorläufigen Erkenntnisse darauf hin, dass a‑tDCS eine signifikante Verbesserung der Benenn- und Kommunikationsfähigkeiten bei chronischer Aphasie nach Schlaganfall bewirken kann im Vergleich zur Sprachtherapie ohne tDCS. Diese Verbesserungen zeigen mittlere bis große Effektstärken und weisen auf die Stabilität der erzielten Behandlungsergebnisse hin.

Auf den bisherigen Erkenntnissen beruhend wurde die Studie Transcranial Direct Current Stimulation to Enhance Training Effectiveness in Chronic Post-Stroke Aphasia (kurz: DC_TRAIN_APHASIA; Transkranielle Gleichstromstimulation zur Verbesserung der Trainingseffektivität bei chronischer Aphasie nach Schlaganfall) initiiert, welche in einem multizentrischen randomisiert-kontrollierten Ansatz klären soll, ob sich die Effektstärke intensiver Sprachtherapie durch den Einsatz von a‑tDCS als therapieadjuvante Applikation erhöhen lässt [16]. In dieser Phase-III-Studie mit 19 Aphasiebehandlungszentren (aktiv 16^1^) in 9 Bundesländern, die im November 2019 gestartet ist und an der Universitätsmedizin Greifswald koordiniert wird, sollen 130 Menschen mit chronischer Aphasie bis Dezember 2024 bundesweit rekrutiert werden.

Direkt nach ihrem Start Ende 2019 war die Studie DC_TRAIN_APHASIA von der Corona-Pandemie betroffen, weshalb es früh zur Herausforderung wurde, eine ausreichende Anzahl Menschen mit Aphasie zu erreichen und zu rekrutieren. Eine Arbeit von Treweek et al. [17] fasst bewährte Rekrutierungsoptionen für randomisiert-kontrollierte Studien zusammen. Die Autor:innen empfehlen u. a. telefonische Erinnerungen bei Teilnehmer:innen, die nicht selbst auf Anfragen reagieren. Als erfolgreich erwiesen sich auch die Verwendung von „Opt-out-“ statt „Opt-in-Verfahren“ (Möglichkeit zur Kontaktaufnahme bei Nichtwiderspruch) und „offene“ Studiendesigns, in denen die Zuteilung zu den Interventionsgruppen bekannt ist [17]. Aus ethischen bzw. methodischen Gründen können letztere Strategien jedoch nicht grundsätzlich empfohlen werden. Ein Review von Houghton et al. [9] benennt Wissenschaftskommunikation, den Einfluss persönlicher Faktoren und des direkten Umfelds sowie den persönlichen Nutzen einer Teilnahme als wichtig für die Rekrutierung. Preston et al. [15] berichten von höheren Rekrutierungsraten beim Einsatz speziell für die Rekrutierung zuständiger und geschulter Personen und eines automatischen Systems zur Identifizierung geeigneter Teilnehmender in Datenbanken. Eine Studie von Patel et al. [14] beschreibt Methoden zur Steigerung der Bekanntheit und Zugänglichkeit von Studien: Sie unterteilt in „Allgemein“ (z. B. Design eines Projektlogos, Erstellung von Visitenkarten und Bereitstellung einer kostenlosen Rufnummer inkl. eines Anrufbeantworters), „Öffentlichkeit“ (z. B. Radiobeitrag, Zeitungsinserate und Information in öffentlichen Gebäuden) und „klinische Gruppen“ (z. B. Präsentationen bei zielgruppenspezifischen oder öffentlich zugänglichen Veranstaltungen). McDonald et al. [12] nennen (a) das Versenden von Newsletters, E‑Mails und Flyern an Klinikpersonal und/oder Patient:innen, (b) regelmäßige persönliche Kontaktaufnahmen mit Kliniken/Praxen und (c) Information mittels Plakaten und Informationsblättern in Kliniken/Praxen als häufigste Rekrutierungsoptionen.

DC_TRAIN_APHASIA untersucht in einem randomisiert-kontrollierten Setting, ob durch a‑tDCS des linken M1-Areals (verglichen mit sham-tDCS) der Effekt einer 3‑wöchigen intensiven Sprachtherapie auf die Kommunikation verstärkt werden kann [16] und ob dieser inkrementelle Nutzen auch für alltagsnahe Kommunikationsleistungen nachweisbar ist. Zur Prüfung alltagsnaher Kommunikation wird der Amsterdam-Nijmegen Everyday Language Test (ANELT) zum Zeitpunkt des 6‑monatigen Follow-ups als primärer Outcomeparameter verwendet. In diesem Beitrag wird das Studienprotokoll vorgestellt sowie die umfangreichen Rekrutierungsstrategien erläutert und diskutiert.

## Material und Methode

### Studienhypothesen

Die Hypothesen und Endpunkte der Studie DC_TRAIN_APHASIA lauten wie folgt [16]:

- *Primäre Hypothese:* Intensive Sprachtherapie führt in Kombination mit a‑tDCS zu einer signifikant besseren Kommunikationsfähigkeit als intensive Sprachtherapie in Kombination mit sham-tDCS
  - *Primärer Endpunkt:* ANELT (Testzeitpunkt t2, 6 Monate nach Therapieende)
- *Sekundäre Hypothese:* Intensive Sprachtherapie führt in Kombination mit a‑tDCS zu besseren Ergebnissen in den sekundären Endpunkten
  - *Sekundäre Endpunkte:* Sprachliche/sprachlich-exekutive Fähigkeiten, Aufmerksamkeit, Gedächtnis, emotionales Wohlbefinden, Lebensqualität und gesundheitsökonomische Kosten (Testzeitpunkte t1 bis t3)

### Studiendesign

**Figure 1:**
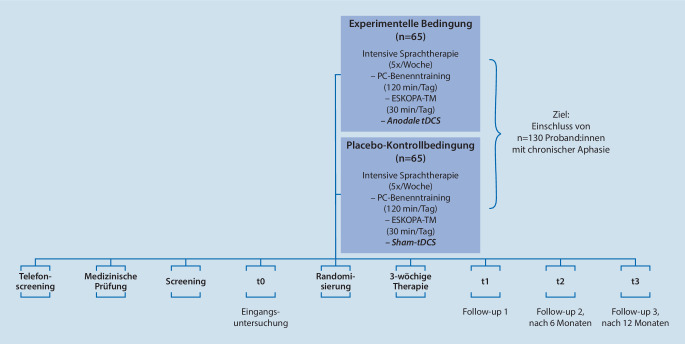

Das Kernstück des Studiendesigns bildet die 3‑wöchige intensive Sprachtherapie (12,5 h pro Woche, d. h. täglich 2,5 h), kombiniert mit a‑tDCS über M1 [16]. Die Therapie besteht ähnlich wie bei Meinzer et al. [13] aus täglich 2 Sitzungen á 60 min eines computergestützten Benenntrainings mit der Studiensoftware AphaApp und a‑tDCS bzw. sham-tDCS. Ergänzend zur Vorstudie [13] kommt innerhalb von DC_TRAIN_APHASIA eine reduzierte Version der „Evidenzbasierten sprachsystematischen und kommunikativ-pragmatischen Aphasietherapie nach ESKOPA-TM“ [8] zum Einsatz (Abb. 1). Zusätzliche Sprachtherapie erfolgt nicht.

### Ein- und Ausschlusskriterien der Population

**Figure 2:**
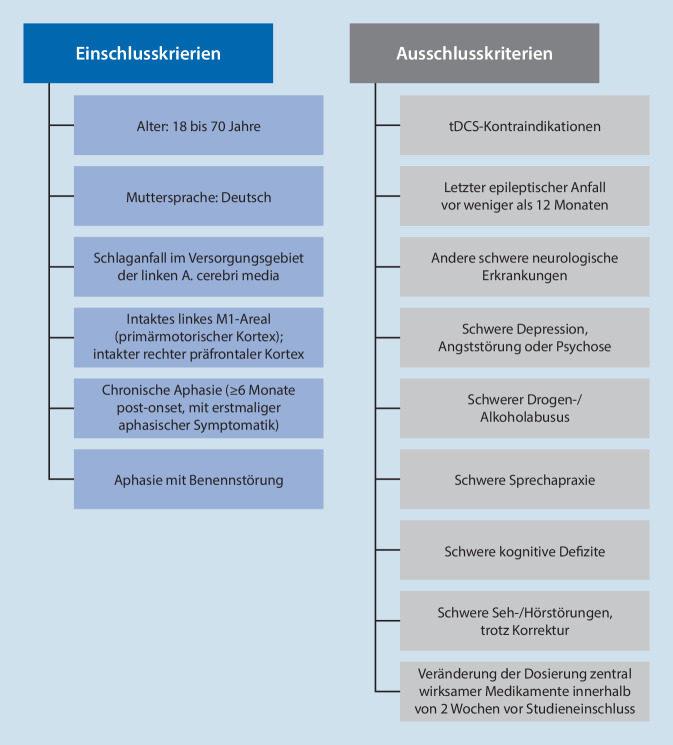

Die erforderliche Zielstichprobe wurde vor Studienbeginn durch die in Abb. 2 dargestellten Ein- und Ausschlusskriterien definiert [10].

Der erste Schritt zur Teilnahme an DC_TRAIN_APHASIA ist ein telefonisches Screening, in dem erste wichtige Ein- und Ausschlusskriterien erfragt werden (Abb. 1). Im Anschluss folgt ein weiteres Screening, das anhand zugesandter Unterlagen bzw. einer Testung vor Ort zusätzliche medizinische und logopädische Ein- und Ausschlusskriterien prüft. Die logopädischen und kognitiven Kriterien sind in Tab. 1 dargestellt.

**Table 1:** 

Einschlusskriterien	Ausschlusskriterien
Chronische Aphasie (≥ 6 Monate post-onset, mit erstmaliger aphasischer Symptomatik)	Schwere Sprechapraxie (Hierarchische Wortlisten; Ausschluss bei > 15 Nullreaktionen mit sprechapraktischer Ursache, bei > 35 Fehlern mit phonematischer Entstellung oder gestörtem Redefluss)
Aphasie mit Benennstörung	Schwere kognitive Defizite (Corsi Block-Tapping Task; Ausschluss bei < 4 korrekt produzierten Würfeln)
→ Aphasiediagnose (Aachener Aphasie Test, AAT)	–
→ Benennstörung (PC-Programm)	–
→ Mind. eine korrekte Reaktion in Teil 1 des AAT-Untertests „Token Test“, d. h. grundlegendes Sprachverständnis	–
→ Mindestens Punktwert 1 im AAT-Untertest „Spontansprache“ (Ebene 1, Kommunikationsverhalten), d. h. grundlegende kommunikative Fähigkeiten	–

### Rekrutierung

Methodisch wurde zunächst mit gängigen Rekrutierungsstrategien begonnen. Aufgrund der Corona-Pandemie, die etwa zeitgleich mit dem Beginn von DC_TRAIN_APHASIA einsetzte, verlief die Rekrutierung unter erschwerten Bedingungen. Ängste der Patient:innengruppe sowie sich ändernde gesundheitspolitische Maßnahmen in den verschiedenen Bundesländern mussten berücksichtigt werden. Die Strategien wurden daraufhin angepasst. Neben Institutionen und Kanälen, über die Patient:innen erreichbar sind, wurde die Rekrutierung im Verlauf ausgeweitet, mit dem Ziel, Angehörige sowie Therapeut:innen und Ärzt:innen der Patient:innen anzusprechen. Unterstützend wurden allen kontaktierten Praxen telefonische Beratungsgespräche zu potenziellen Proband:innen angeboten, was gern genutzt wurde. Eine Auflistung aller vielfältigen angewandten Rekrutierungsmethoden findet sich in Tab. 2:

**Table 2:** 

Bereich	Rekrutierungsmaßnahme
Institutionen	Studienzentren
	Ca. 700 logopädische Praxen deutschlandweit (Übersichten: https://www.dbl-ev.de/service/logopaedensuche und https://www.dbs-ev.de/service/therapeutenverzeichnis/)
	Rehabilitationseinrichtungen
	Neurologische, hausärztliche, neurochirurgische und radiologische Praxen
	Neuropsychologische, ergotherapeutische und physiotherapeutische Praxen
	Logopädieschulen deutschlandweit
	Logopädiestudiengänge inkl. Verteiler
	Logopädische Fortbildungsinstitute
	Apotheken
	Ausgewählte Einwohnermeldeämter und Rathäuser
	Institutionen zur beruflichen Wiedereingliederung
	Pflegeheime/-dienste
Kostenträger	Krankenkassen → Newsletters und Homepage
Patient:innenverbände und Selbsthilfe	Bundesverband Aphasie e. V.
	Landesverbände Aphasie (https://aphasiker.de/landesverbaende/)
	Regionalgruppen Aphasie, u. a. Berliner Aphasikerchor
	Stiftung Deutsche Schlaganfall-Hilfe → Newsletters
	Berliner Schlaganfall-Allianz
Berufs‑/Fachverbände	Deutscher Bundesverband für Logopädie e. V. → Website und Mitgliederportal
	Deutscher Bundesverband für akademische Sprachtherapie und Logopädie e. V. → Website
	Gesellschaft für Aphasieforschung und -behandlung e. V. → Jährliche Mitgliederversammlung
	Deutsche Gesellschaft für Elektrostimulation und Elektrotherapie e. V. → Netzwerk innerhalb der tDCS-Community → Newsletter
Vorträge	Jahreskongress des Deutschen Bundesverbandes für Logopädie e. V.
	Landesverbandssitzungen des Deutschen Bundesverbandes für Logopädie e. V.
	Virtuelle Würzburger Aphasie-Tage – Vorträge, Workshop und virtueller Stand
	Neurowoche der Deutschen Gesellschaft für Neurologie e. V.
	Kongress der Deutschen Gesellschaft für Neurorehabilitation e. V.
	Webinar der Universitätsmedizin Greifswald zum Weltschlaganfalltag
	Gruppentreffen der Aphasieselbsthilfe
	Sonstige wissenschaftliche Vorträge – jeweils am Ende „Werbung“
Fernsehen und Radio	Bericht NDR Visite im April 2021 → Beitrag „Hirnstimulation: Behandlung mit Strom und Magnetfeldern“ (https://www.ndr.de/ratgeber/gesundheit/Hirnstimulation-Behandlung-mit-Strom-und-Magnetfeldern,hirnstimulation101.html)
	Radiobeitrag NDR Info
	Eigener Werbespot bei privatem Berliner Radiosender
Print(‑Medien)	Regionale Tageszeitungen → Z. B. Ostseezeitung und Filstalexpress
	Logopädische Fachzeitschriften → Z. B. Sprache Stimme Gehör
	Medizinische bzw. neurologische Fachzeitschriften → Z. B. Gesundheitsmagazin Thala der Stiftung Deutsche Schlaganfall-Hilfe
Internet und Social Media	Studienhomepage inkl. eines Logos (https://aphasie-hirnstimulation.de/)
	Facebook- (DC Train Aphasia) und Instagram-Account (@dc_train_aphasia) → Interaktion mit Studieninteressierten, Kliniken und Studienzentren → Facebook: Information von Aphasie- und Therapeut:innengruppen → Instagram: Kontaktierung logopädischer Praxen, neurologischer Expert:innen und „Influencer:innen“ im Bereich Logopädie
	Verlinkung der Studie auf Websites und Social-Media-Kanälen der Studienzentren
	Logopädische Online-Blogs/Apps
	Kleinanzeigenportale
	Podcast aus dem Bereich Neurologie
Rekontaktierung	Vorherige Aphasiestudien (bei Einwilligung)
	Hausinterne Patient:innendatenbank (bei Einwilligung^a^)
Weiteres	Brainstorming, Studienteam/-beteiligte
	Forschungskolloquien der Studienzentren
	Interesse potenzieller Teilnehmender wecken mittels a‑tDCS (als Adjuvanz zur Regelversorgung bei Aphasie)

^a^Die Teilnehmenden hatten einer Rekontaktierung zuvor ausdrücklich zugestimmt und das Vorgehen wurde durch die zuständige Ethikkommission sowie den Datenschutz genehmigt

Wie aus der Literatur bekannt, ist der persönliche Kontakt bei der Rekrutierung entscheidend [11]. Der telefonische Erstkontakt ist ein Informationsgespräch, das bei grundsätzlichem Interesse meist unmittelbar durch ein telefonisches Screening erweitert wird (Abb. 1). Das Gespräch wird von Sprachtherapeutinnen der DC_TRAIN_APHASIA-Studienzentrale geführt, die geschult sind in „unterstützter Kommunikation“. Fragen der Interessierten werden in verständlicher Weise beantwortet. Außerdem wird ausführlich auf mögliche Vorteile der Therapie für Teilnehmende, die wichtigsten wissenschaftlichen Grundlagen, die Bedeutung einer Mitwirkung für die klinische Versorgung sowie Risiken und Nebenwirkungen des Verfahrens hingewiesen.

### Studiendurchführung

Die Behandlung wird auf den individuellen Sprachstand jedes Studienteilnehmenden zugeschnitten. Für die Benenntherapie werden mithilfe des Programms AphaApp beim logopädischen Screening individuelle Items ausgewählt [13]. Ebenso werden anhand des Screenings für die „Evidenzbasierte sprachsystematische und kommunikativ-pragmatische Aphasietherapie nach ESKOPA-TM“ [8] individuelle Therapieschwerpunkte bestimmt. So werden mit allen Studienpatient:innen gezielt verbale und nonverbale Alltagssituationen des individuellen Schwierigkeitsgrades im Kontext der sozialen Interaktion trainiert [8].

Die Sprachtherapie wird mit a‑tDCS bzw. sham-tDCS kombiniert. In der Interventionsgruppe erfolgt zu Beginn jeder Sitzung eine 20-minütige a‑tDCS über M1 (pro Tag 2 × 20 min, 1 mA). In der Kontrollgruppe wird der Strom für 30 s lang auf 1 mA hochgefahren, bevor er bis zum Ende der 20-minütigen Scheinstimulation heruntergefahren wird [13, 16]. Die Gruppenzuordnung im Verhältnis 1:1 wird durch die Studienzentrale der Universitätsmedizin Greifswald stratifiziert-randomisiert (Blockrandomisierung). Da es sich um eine Doppelblindstudie handelt, gibt ein batteriebetriebener tDCS-Stimulator (DC-Stimulator Plus, NeuroConn, Ilmenau, Deutschland) im „Studienmodus“ die entsprechende Stimulation im Therapiesetting aus [16]. Die Anode (5 × 7 cm^2^) wird waagerecht über M1 links (C3-Elektrode des 10–20 EEG-Systems) positioniert. Die Kathode (10 × 10 cm^2^) wird über der rechten supraorbitalen Region platziert [13].

## Zwischenergebnisse

### Rekrutierung und Studientherapie

Aktuell wurden 159 Studieninteressierte gescreent, von welchen 110 Patient:innen in die Studie DC_TRAIN_APHASIA eingeschlossen wurden. Entsprechend konnten 49 Proband:innen nicht eingeschlossen werden. Beim Screening war der Ausschluss bislang zumeist bedingt durch zu schwere Beeinträchtigungen im Aachener Aphasie Test (AAT), eine nicht zuverlässig nachweisbare Restaphasie (AAT) oder eine zu gering ausgeprägte Benennstörung (laut computerbasierter Diagnostik mit der AphaApp). Ein medizinischer Ausschluss war am häufigsten auf eine zu große Schädigung des linken M1-Kortex laut aktueller Bildgebung (CT oder MRT) zurückzuführen.

Im Sommer 2022 kam ein Studienzentrum in Rostock hinzu. In den 19 teilnehmenden Zentren haben bislang 89 Patient:innen die Therapie durchlaufen. Dementsprechend bestand trotz der großen Herausforderung der Corona-Pandemie, die von Anfang 2020 bis Ende 2022 andauerte, über die letzten 3,5 Jahre ein kontinuierlicher Fortschritt. Die Rückmeldungen zur Studienteilnahme in den Zentren sowie zur Organisation waren bisher sehr positiv. Derzeit ist geplant, noch bis Ende 2024 zu rekrutieren und Therapien durchzuführen. Mit ersten Ergebnissen ist ab Mitte 2025 zu rechnen.

### Evaluation Rekrutierungsmethoden

Dass die Studie zu keinem Zeitpunkt pausierte, sondern kontinuierlich weiterlief, ist auch auf die großen Bemühungen bei der Rekrutierung zurückzuführen. Methoden, die besonders viel Rücklauf generierten, waren die Kontaktierung von Selbsthilfegruppen, Logopäd:innen und Neurolog:innen, weshalb diese regelmäßig wiederholt werden. Der Fernseh- und Radiobeitrag zur Studie zeigte sich als kurzfristig sehr erfolgreich, v. a. direkt nach der Ausstrahlung und solange die Beiträge in der Mediathek des NDR online abrufbar waren. Soziale Medien können zunehmend zur Rekrutierung beitragen. Um einen Einfluss der Rekrutierungsmethoden auf die Repräsentativität im Hinblick auf die Zielpopulation gering zu halten, wurde versucht, sowohl Patient:innen als auch Angehörige und Therapeut:innen zu erreichen.

### Therapiephase und tDCS-Sicherheitsaspekte

Das Data Safety and Monitoring Board, welches regelmäßig tagt und die Sicherheit der klinischen Phase-III-Studie anhand aufgetretener „unerwünschter Ereignisse (‚adverse events‘, AEs)“ beurteilt, äußerte bislang keine Sicherheitsbedenken, sodass das Studiendesign unverändert blieb. Außer typischen Nebenwirkungen wie leichte Kopfschmerzen, Kribbeln oder Brennen unterhalb der Elektroden, die bei a‑tDCS auftreten können [1], wurden keine schwerwiegenderen Nebenwirkungen berichtet. „Schwerwiegende unerwünschte Ereignisse (‚severe adverse events‘, SAEs)“ traten glücklicherweise nicht auf.

### ANELT und Endpoint Committee

Das Endpoint Committee ratet randomisiert je Teilnehmer:in in variablen Zweierteams den ANELT als primären Outcomeparameter der Studie. Die Beurteiler:innen sind verblindet bez. der Gruppenzuordnung und des Messzeitpunktes der ANELT-Aufnahme. Vor Beginn der Ratings konnte eine zufriedenstellende Interraterreliabilität über die 8 Beurteiler:innen erreicht werden.

### Weiteres

Neben dem Data Safety and Monitoring Board und dem Endpoint Committee ist das Trial Steering Committee ein wichtiges Gremium der Studie. Es hält regelmäßig Sitzungen ab, bei welchen dem Studienteam der Einbezug von Patientenvertreter:innen wichtig ist. Sowohl dort als auch bei regelmäßigen Get-Togethers, bei welchen alle Studienbeteiligten zusammenkommen, ist die Meinung von Betroffenen entscheidend und wird mit hoher Priorität berücksichtigt. Zur statistischen Auswertung ab 2025 liegt ein detaillierter Datenanalyseplan vor.

## Diskussion

Die multizentrische Phase-III-Studie DC_TRAIN_APHASIA, die a‑tDCS als therapieadjuvante Applikation untersucht, rekrutiert noch bis Ende 2024. Die Studie fiel unmittelbar in den Zeitraum der Corona-Pandemie. Im Vordergrund stand daher während der Pandemie vor allem, bei der Rekrutierung auf die individuellen Ängste und Bedürfnisse der Patient:innen einzugehen. Hierbei ist ein sensibles Vorgehen der involvierten Mitarbeiter:innen erforderlich [15]. Wie von Houghton et al. [9] empfohlen, war insbesondere die umfangreiche Darstellung und Diskussion der Vor- und Nachteile einer Teilnahme an klinischen Studien wichtig, um Proband:innen und ihre Angehörigen zu überzeugen. Automatische Systeme zur Identifizierung passender Proband:innen [15] in vorliegenden Datenbanken stellten sich als hilfreich heraus. Auch von Treweek et al. [17] vorgeschlagene telefonische Erinnerungen und das persönliche Gespräch waren obligatorisch und effektiv. Der Einsatz von „Opt-out-Verfahren“ hingegen war aus datenschutzrechtlichen Gründen in der Studie DC_TRAIN_APHASIA nicht möglich. Ebenso war es im Rahmen der vorliegenden Studie aufgrund der Anlegung als Doppelblindstudie nicht möglich, die Gruppenzuordnung vorab bekanntzugeben. Die von McDonald et al. [12] und Patel et al. [14] beschriebenen Methoden wie der Kontakt zu Kliniken/Praxen sowie Radiobeiträge, Zeitungsinserate und Information in öffentlichen Gebäuden konnten erfolgreich umgesetzt werden.

Die in diesem Beitrag vorgestellten Rekrutierungsstrategien vermitteln einen Eindruck der vielfältigen Herausforderungen bei der Durchführung einer groß angelegten multizentrischen Aphasiestudie. Durch die Zusammenstellung wird Studien ein Leitfaden für die Rekrutierung zur Verfügung gestellt, sodass klinische Aphasiestudien im deutschsprachigen Raum, aber auch darüber hinaus, zukünftig erfolgreicher rekrutieren können. Zur Stärkung der Rekrutierung durch die klinischen Partner könnte ein „Siegel-System“ eingeführt werden. Kliniken könnten dieses „Forschungssiegel“ verwenden, um ihr Engagement in der klinischen Forschung nach außen sichtbar darzustellen und so für ihre Einrichtung zu werben.

## Fazit für die Praxis

Der vorliegende Artikel beschreibt die derzeit Teilnehmer:innen rekrutierende multizentrische randomisiert-kontrollierte Studie DC_TRAIN_APHASIA und ihren aktuellen Stand. Es werden weitere Proband:innen mit chronischer Aphasie für die Studie gesucht. Die vorgestellten Methoden zur Rekrutierung haben die erfolgreiche Fortführung der Studie während der Corona-Pandemie ermöglicht. Die Übersicht soll künftigen klinischen Studien als Leitfaden und Checkliste dienen zur Rekrutierung und Durchführung, insbesondere bei Proband:innen mit eingeschränkten kommunikativen Fähigkeiten.

## Footnotes

1 Uniklinik RWTH Aachen; Kliniken Schmieder Allensbach, Gailingen und Heidelberg; Wicker Klinik Bad Homburg; Moritz Kliniken Bad Klosterlausnitz; Medical School Berlin (Lehr- und Forschungsambulanz für Psychotherapie bei Aphasie); Klinikum Christophsbad Göppingen; Universitätsmedizin Greifswald; Hospital zum Heiligen Geist Kempen; Tagesklinik Max-Planck-Institut Leipzig; LogoZentrum Lindlar; TheraVent aktiv Marbach; St. Mauritius Therapieklinik Meerbusch; Europäische Fachhochschule (EUFH) Rostock; Aphasie Zentrum Josef Bergmann Vechta.
